# Supplementary material for: Patterns of Intron Gain and Loss in Fungi
Source: PLoS Biol. 2004 Nov 30;2(12):e422. doi: 10.1371/journal.pbio.0020422 (PMC532390; doi:10.1371/journal.pbio.0020422)
Supplement: Table S1 — Also available at http://genes.mit.edu/NielsenEtAl/. (4.3 MB ZIP). [file pbio.0020422.st001.zip › NielsenEtAl/html/1439.html]

AN4707.1.NCU00664.1.MG02820.1.FG00188.1


```
 CLUSTAL W (1.82) Multiple Sequence Alignments - Introns Inserted


Sequence 1: NCU00664.1	239 aa
Sequence 2: MG02820.1	246 aa
Sequence 3: FG00188.1	243 aa
Sequence 4: AN4707.1	256 aa
Alignment Length: 257 aa
Number Identitical Residues: 147 aa
Alignment Score (without introns) 6916


MG02820.1 	M0ARNSEKAQSMLFRFREAQAADLGIIDAGRTRRPRAITEQDSIPACEKWRGQVLKDISR
NCU00664.1	M0ARNSEKAQSMLFRFREAQAADLGIIDAGRTRRPRSITEQDSIPACEKWRGQVLKEISR
FG00188.1 	M0ARNSEKAQSMLFRFREAQAADLGIIDAGRTRRPKLITEVTTIPACEKWRGQVLKEISR
AN4707.1  	M0ARNSEKAQSMLFRFRAQQAADLGIIDIGRTRRPKAITSVDSIPACEKWRGQVLKEISR
          	* ***************  ********* ******: **.  :*************:***

MG02820.1 	KVSRIQESSLSDYQIRDLNDEINKLMREKHMWEVQIRNLGGPNYTRAGGAKVYDEAGKEI
NCU00664.1	KVSRIQDPALSDYQIRDLNDEINKLMREKHMWEVQIRNLGGPNYMRSGG-KVYDEAGREI
FG00188.1 	KMSRIQEPILSDYQIRDLNDEINKLMREKHMWEIQIRNLGGPNYMRGGG-KIYDDQGREI
AN4707.1  	KVSRIQEPSLSDHQIRDLNDEINKLMREKWAWEMQIRNMGGPNYMRGSG-RVYDDEGREI
          	*:****:. ***:****************  **:****:***** *..* ::**: *:**

MG02820.1 	GGAGRGYRYFGRAKELPGVKELFEAA-R-------AKKGDEKPLEARDDLR-KQVDAAYY
NCU00664.1	PGGGRGYKYFGRARELPGVKELFEAAAR-------AKQDDEKPLETRDDLR-KQVDAAYY
FG00188.1 	PGSGKGYKYFGRARELPGVKELFEAA---------KNQGDEKPLEERHDMR-RNVDAAYY
AN4707.1  	PGGGKGYRYFGRARELPGVKEMLEAAARRGRGPAEEEEDDGKTVGRGGDIATKKVDANYF
          	 *.*:**:*****:*******::***:  . ..:. ::.* *.:    *: :::*** *:

MG02820.1 	GYSPDEEDDELLAYEAAKEKEAFENLADNRKG-EQLPPGWEPLPGDNGDGKGWELPTVEE
NCU00664.1	GYAPDEEDEKLLAYEAARERQAFENLAKAAAG-LEPPPGWEPLP-------EWELPTMDE
FG00188.1 	GYAPDEEDEELLAYEAEKERQAVDHMLKTGP--RDVPDGWEALPGDTGDGNTWVLPTLEE
AN4707.1  	GYGLDEEDGTLLAYEMQREKEAVEKLRKEGEDEEDVEDGWEPLPGDAGDGIEWRLPTLEE
          	**. ****  *****  :*::*.::: .   .. :   ***.**.. ...  * ***::*

MG02820.1 	VQQELINRRKQRLLNQL-
NCU00664.1	VAQELIDRRRRRLLDQL-
FG00188.1 	VQEELIERRRRKLLEQL-
AN4707.1  	VQEELVDRRRRRLLEKIS
          	* :**::**:::**::::
```
